# Types and frequency of errors in the preparation and administration of drugs

**DOI:** 10.1590/S1679-45082018AO4146

**Published:** 2018-09-10

**Authors:** Josiane Ribeiro Mendes, Maria Carolina Barbosa Teixeira Lopes, Cássia Regina Vancini-Campanharo, Meiry Fernanda Pinto Okuno, Ruth Ester Assayag Batista

**Affiliations:** 1 Universidade Federal de São Paulo, São Paulo, SP, Brazil

**Keywords:** Emergency nursing, Patient safety, Medication errors, Administration, intravenous, Enfermagem em emergência, Segurança do paciente, Erros de medicação, Administração intravenosa

## Abstract

**Objective:**

To identify compatibility, types and frequency of errors in preparation and administration of intravenous drugs.

**Methods:**

A cross-sectional and descriptive study performed at the emergency department of a university hospital in the city of São Paulo (SP). The sample consisted of 303 observations of the preparation and administration of intravenous drugs by nursing aides, nursing technicians and registered nurses, using a systematized script, similar to a checklist. The following variables were collected: errors related to dispensing, omission, schedule, unauthorized administration, dosage, formulation, incompatibility, preparation and administration.

**Results:**

In the preparation stage, the following errors were identified: no hand hygiene (70.29%), and no use of aseptic technique (80.85%). Upon administration, no hand hygiene (81.18%), and no use of aseptic technique (84.81%). In 31.35% of observations, there was more than one medication at the same time for the same patient, of which 17.89% were compatible, 56.84% were incompatible and 25.26% were not tested, according to the Micromedex database.

**Conclusion:**

In both preparation and administration stages, the most frequent errors were no hand hygiene and no use of aseptic technique, indicating the need to develop and implement education programs focused on patient safety.

## RESUMO

**Objetivo:**

Identificar a compatibilidade, os tipos e a frequência de erros no preparo e na administração de medicamentos endovenosos.

**Métodos:**

Estudo transversal e descritivo, realizado em um serviço de emergência de um hospital universitário da cidade de São Paulo (SP). A amostra foi constituída por 303 observações do preparo e administração de medicamentos endovenosos por auxiliares, técnicos de enfermagem e enfermeiros, tendo como instrumento um roteiro sistematizado, do tipo *checklist.* As variáveis coletadas foram: erro de dispensação, omissão, horário, administração não autorizada, dose, apresentação, incompatibilidade, e erros de preparo e administração.

**Resultados:**

Na etapa de preparo, foram identificados os seguintes erros: não higienização das mãos (70,29%) e não usar técnica asséptica (80,85%). Na etapa de administração, 81,18% não higienizaram as mãos, e 84,81% não usaram a técnica asséptica. Em 31,35% das observações, existia mais de uma medicação no mesmo horário para o mesmo paciente; destas 17,89% eram compatíveis, 56,84% incompatíveis e 25,26% não foram testadas, conforme análise feita na base de dados Micromedex.

**Conclusão:**

Tanto na etapa do preparo quanto na de administração, os erros mais frequentes foram a não higienização das mãos e o não uso de técnica asséptica, apontando para a necessidade de desenvolvimento e implantação de programas de educação centrados na segurança do paciente.

## INTRODUCTION

Emergency departments are settings specialized in care of patients with acute clinical picture, which might be life-threatening conditions.^(^ ^1^ ^)^ Overcrowding in these departments is a worldwide phenomenon, resulting mainly from inadequacy of the demand due to insufficient structuring of the healthcare network, in addition to the increased number of accidents and urban violence.^(^ ^2^ ^)^

Over the past decades, overcrowding at emergency departments has been associated with worsening of quality of care, due to stress, absenteeism, and lack of professionals at these sites; to high patient turnover rate, and excessive workload, which can lead to adverse events.^(^ ^3^ ^,^ ^4^ ^)^

The prevention of adverse events, with the consequent increase of patient safety, gained greater visibility after the publication of “To err is human: Building a safer health system”, by the Institute of Medicine (IOM), in 1999. In this work, data related to the number of healthcare-related deaths in the United States were presented, drawing attention of governmental organizations to quality of care and patient safety.^(^ ^5^ ^)^

A culture of safety is a set of values, competences and behaviors, which determine the commitment to management of health and safety; hence, it replaces guilt and punishment by the opportunity to learn from failures and improve healthcare.^(^ ^6^ ^)^

Patient safety is a serious public health problem all over the world. In developed countries, it is estimated that one in every ten patients is harmed while receiving care at hospital. Amidst this care, administration of medications can pose risks to the individual's safety.^(^ ^5^ ^)^

A medication error is defined as “any avoidable event that can cause or induce the inappropriate use of medication or harm the patient, at any phase of drug therapy. ”^(^ ^7^ ^)^ Medication errors are among the most frequent failures in healthcare and can cause complications in the clinical picture, require new interventions, increase length of hospital stay, or even permanent disabilities and death.^(^ ^5^ ^)^

A study conducted in the United States demonstrated that every patient admitted to a North American hospital is subject to one medication error a day. Approximately 400 thousand avoidable medication-related adverse events are reported per year at these sites.^(^ ^8^ ^)^ It is estimated that medication errors in hospitals cause more than 7,000 deaths per year in the United States, leading to significant costs.^(^ ^9^ ^)^

In Brazil, statistics as to deaths by medication errors are still scarce. Data from the *Instituto para Práticas Seguras no Uso de Medicamentos* (ISMP) [Institute for Safe Practices in Medication Use] showed that at least 8,000 deaths per year are attributable to medication errors, in which failures or adverse reactions resulting from administration of medications corresponded to 7.0% of admissions to the health system, accounting 840 thousand cases/year.^(^ ^10^ ^)^

Promoting safe practices in administration of medications should be a constant concern of the nursing team, since it is one of the tasks more often performed by these professionals.^(^ ^11^ ^)^ Some factors, such as insufficient quantity of professionals, excess work, exhausting working hours, lack of materials, high number of medications to be given, interruptions during preparation and administration of medications, precarious lighting, and excessive noise predispose towards the occurrence of errors.^(^ ^10^ ^)^

Even if many medication errors do not cause serious consequences to patients, they should be notified and studied to avoid their recurrence, and to strengthen a safe drug administration system.^(^ ^12^ ^)^ Identification of medication errors is fundamental, since it supports the decisions necessary to avoid them. The presence of a punitive culture towards the professional who made the error leads to underreporting, reflects this responsibility is not shared between the employee and the organization, which should foresee and offer adequate working conditions for its team.^(^ ^13^ ^)^

The emergency department, due to the dynamics of its care, is characterized by some factors, such as stress and the scarcity of professionals. It is considered a high-risk area for the occurrence of medication-related adverse events, such as adverse reactions, interactions, allergic reactions, and medication errors. The risk potential for medication errors in this department is observed, primarily, by the quantity of medications prescribed and administered by various routes including intravenous route, which requires the addition of electrolytes and the calculation of drip rates during critical phases of care. However, this risk can occur in other phases of the medication process, and even worsen due to the quantity of patients seen at these sites.^(^ ^14^ ^)^

## OBJECTIVE

To identify types and frequency of errors in the preparation and administration of intravenous medications at an emergency department.

## METHODS

This was a cross-sectional and descriptive study, approved by the Research Ethics Committee of the *Universidade Federal de São Paulo,* under official opinion number 1.463.028, CAAE number: 52035115. 0.0000.5505.

The study was conducted at the emergency department of *Hospital São Paulo* (HSP), a large high-complexity tertiary hospital that mainly cares for patients from the *Sistema* Único *de Saúde* (SUS) [Brazilian National Health System].^(^ ^15^ ^)^

The convenience sample was composed of opportunities for preparation and administration of intravenous medications, performed by the emergency department nursing professionals, during the data collection period.

The professionals authorized participation in the investigation before the start of data collection, and the professional did not know when the observation would occur.

Data collection was performed from April to September 2016, in the four work shifts of the nursing team, by means of a structured instrument for observation with the following variables: category of professional who administered the medication, drug class and expiry date of medications, and errors found in the process of preparation and administration of the drugs.

In this study, the errors were classified according to clinical severity as serious or mild. The mild incidents had a small or no effect on the patient, and serious errors caused a reduction or permanent loss of organ function of the patient, and those that lead to death.^(^ ^16^ ^)^

As to the drug therapy phase, the errors were classified as^(^ ^17^ ^)^ dispensing error - when there was incorrect distribution of the medication prescribed; omission error - when the medication prescribed was not administered, or there was no record of administering medication; time error - when the medication was administered out of the time interval established by the organization; non-authorized administration of the medication - in case of administration of non-prescribed drug, to the wrong patient, or wrong medication, or use of an outdated prescription; dosage error - in cases of administration of a dose greater or smaller than what is prescribed; formulation error - when the medication formulation prescribed was different from what was administered; preparation error - when there were failures in dilution, inadequate storage, failure in asepsis technique, incorrect identification of the drug, and inappropriate choice of infusion lines; and administration errors - such as failure in asepsis technique of the venous lines and connecting devices, administration through a route other than that prescribed, incorrect infusion rate, administration of medication that is expired or with compromised physical or chemical integrity, and association of medications that are physically or chemically incompatible.

In order to verify incompatibility among medications, the Truven Health Analytics, Micromedex (Greenwood Village, Colorado, United States) software was used. Since dipyrone is not a part of this software and was one of the most medications more often observed in this study, its incompatibility was analyzed according to information contained on the package insert.

Due to ethical and patient safety issues, when the investigator identified a potential or real error, observation was immediately interrupted and the investigator intervened by means of orientation of the employee as to the correct practice.

### Statistical analysis

Descriptive analysis was used. For continuous variables, the mean, standard deviation, median, minimum and maximum values were calculated; for categorical variables, frequency and percentage.

## RESULTS

The study sample comprised 303 observations of preparation and administration of intravenous medications. The professional categories observed were 60.0% nursing aides, 32.6% nurse technicians, and 7.2% registered nurses.

The drug classes administered by these professionals were antimicrobials (24.7%), non-opioid analgesics (23.1%), anti-inflammatory agents (10.5%), anti-emetics (9.5%), opioid analgesics (8.9%), antacids (5.6%), antiarrhythmic agents (3.6%), diuretics (3.3%), anticonvulsants (2.9%), vasodilators (1.6%), antispasmodic agents (1.3%), cardiotonic agents (0.9%), splenic vasoconstrictors (0.6%), antidiabetics (0.6%), vasopressors (0.6%), vitamins (0.3%), and bone catabolism inhibitors (0.3%).

All medications administered during the observations were within the expiry period.

As to the medication errors, no note was made of omission errors, non-authorized medication administration, or formulation and dispensing. Regarding dosage errors, 2.6% of medications were administered at a dose higher or lower than the dose prescribed. As to timing errors, 5.6% of medications were not given, respecting the recommendation of not exceeding 30 minutes more or less relative to the time at which the medication was scheduled.

During the preparation of medications, the following errors were identified: lack of hand hygiene before preparation (70.2%); not using an aseptic technique for preparation (80.8%); incorrectly identification of the medication (47.9%); not checking the patient's identification (62.3%), and dilution of the medication in a volume smaller than that recommended by the manufacturer (1.6%).

In the administration stage, the identified failures were no hand hygiene before administration (81.1%); not using an aseptic technique in administration (84/8%), and incorrect administration infusion rate (4.0%). Of the total of medications administered, in 10.8%, checking the pulse rate was recommended, but in 24.3% of the times, this measure was not taken.

In 31.3% of observations, there was more than one medication for the same timepoint and patient: 56.8% of them were incompatible, 25.2% had not yet been tested, and 17.8% were compatible.

## DISCUSSION

Error in the process of preparation and administration of medications can result in serious consequences to the patient and their family, besides generating disabilities, prolonging hospital stay and recovery, demanding new procedures and interventions, delaying or impeding the patient of reassuming their social function, and even leading to death.^(^ ^18^ ^)^

In this study, most opportunities of preparation and administration of medications were of antimicrobials and non-opioid analgesics; the most observed and most prone to error professional category was that of nursing aides. A study conducted at the emergency department of the State of Paraná, with the objective of characterizing the administration of injectable medications, identified that the majority of prescriptions was of analgesic (29.1%), followed by anti-inflammatory agents (26.3%).^(^ ^19^ ^)^ The prescription of analgesics of any drug class that have the relief of pain as secondary effect, is common in emergency departments and first aid units since pain is the most frequent complaint at these places; this fact could justify the finding.^(^ ^20^ ^)^ The fact of the most observed professionals in this study having been the nursing aides, corroborates the literature findings, since the preparation and administration of medications are activities that can be shared among the nursing team and are usually delegated to these professionals.^(^ ^21^ ^)^

In this study, the administration of medications at a dose higher or lower than that prescribed was noted. In most cases, dosage errors can be attributed to the writing of medical prescriptions, such as the use of acronyms and/or abbreviations, absence of the patient's registration, lack of dosing schedule, and omission of the date. Administration of incorrect doses can result in ineffective treatment, prolonged hospital stay and compromise quality of care delivered.^(^ ^22^ ^)^

As to timing errors, in this study, medications were administered outside the recommended timepoint. Brazilian studies have found alarming data relative to this finding: 57.2% of medications were prepared more than one hour before their administration,^(^ ^7^ ^)^ and up to 69.7% of medications were administered at the wrong time.^(^ ^23^ ^)^ Often, the preparation and administration of medications happen at incorrect times due to the frequent practice of optimizing or advancing the activities, which should be supervised and deterred, since efficacy of medications can be compromised when diluted and not immediately administered. Moreover, they are exposed to contamination, light, heat, and humidity. Another relevant factor is the time and duration of action of the drugs, which can be impaired when not given at the correct time, compromising the patient's recovery.^(^ ^24^ ^)^

In this investigation, during preparation and administration of medications, we frequently identified failures in hand hygiene of professionals and in asepsis of materials. A descriptive study aiming to verify and characterize errors in the administration of antibiotics at an intensive therapy unit of a Brazilian teaching hospital, identified absence of disinfection of vials of medications to be given by intravenous route in 58.4% of the times, followed by lack of hand hygiene in 29.2% of nursing team professionals before doing the procedure.^(^ ^24^ ^)^ This individual measure is simple, low-cost, and prevents spread of healthcare-related infections.^(^ ^24^ ^)^

Incorrect identification of the medication, and not checking the patient's identification before administration of medication were errors found in the observations of this study. These two steps - confirming if the patient is correct and the medication correct - are crucial for safe administration of medications.^(^ ^25^ ^)^ The findings of this research can be attributed to the fact of being carried out at an emergency department, a site where the demand for care often exceeds the physical, human resource and material capacities. Additionally, due to the serious status of the patient and risk of death, many medications need to be given rapidly, even with a verbal prescription. Despite this scenario, strategies should be implemented, such as double-checking by the medical and nursing teams, so that safety in medical treatment is improved at these locations.

In this study, the dilution of medication at volume lower than recommended by the manufacturer was identified. A study conducted in a public hospital in the interior of the state of São Paulo, with the objective of identifying the frequency of errors that occurred in the dilution process of intravenous medications, in a critical care unit, showed that out of 180 doses, 125 (69.5%) presented with at least one dilution error, and recording, evaluation and/or monitoring of these errors were not detected.^(^ ^26^ ^)^ These failures should be identified and corrected, such as with educational and reference materials, since these failures can compromise the therapeutic efficacy of said medications.

Another important aspect regarding safety in administration of medications is infusion rate, which in this investigation, was incorrect in some observations. In a literature review aiming at investigating the scientific production on medication administration errors in nursing care practice, the main medication error was associated with the incorrect infusion rate.^(^ ^27^ ^)^ When using the infusion pump, this finding can be associated to programming errors or inadequate handling. When gravitational infusion was used, incorrect drip calculation and lack of supervision by part of the team were noted, indicating the need to train professionals involved in this practice.

In 10.8% of medications administered, checking patient's pulse was recommended, but this measure was not taken in 24.3% of cases. Some medications have the potential of causing modification in vital signs, worsening the patient's health status. In this way, the good practices in administration of medications include ongoing vigilance and monitoring of patients at risk, which could be emphasized at the emergency department, site of our study, since the patients are already, in most cases, in a severe state of health.^(^ ^25^ ^)^

In 31.3% of the observations, there was more than one medication at the same time for the same patient, as to the compatibility of medications that were administered concomitantly during the collection of data of this study, more than half were incompatible and one fourth had not yet been tested. Because of the clinical conditions of patients at the emergency department, treatment with intravenous medications is common, and can result in numerous risks, due to complexity of intravenous pharmacotherapy, especially administration of the medication.^(^ ^28^ ^)^

Incompatibility is an unexpected physical and/or chemical interaction between two or more substances when in a mixture, which can compromise the safety and efficacy of treatment with the product formed. Additionally, incompatibility can imply consequences that go from a simple obstruction of a catheter to an individual's death. It is necessary that the multiprofessional team be attentive to this problem, primarily due to the lack of knowledge and the lack of training of the professionals.^(^ ^28^ ^)^

This study has the limitation the fact of having been conducted at a single center, with a reduced sample size, which could hinder the comparison with other realities and generalization of the results. In addition, the intervention of the investigator, in case of potential or real error, could have caused interference in the findings of the research. Nevertheless, the results of this study can contribute so that individual failures or those in the process of preparation and administration of medications are identified, and that measures for prevention are implemented, such as in training and permanent education of the team, which results in increased quality of care and safety of the patient.

## CONCLUSION

The primary errors related to preparation and administration of intravenous medications were lack of hand hygiene in the preparation and administration of medications, and lack of asepsis of the materials used for the infusion.
